# Distinguishing Between Nile Tilapia Strains Using a Low-Density Single-Nucleotide Polymorphism Panel

**DOI:** 10.3389/fgene.2020.594722

**Published:** 2020-12-01

**Authors:** Matthew G. Hamilton, Curtis E. Lind, Benoy K. Barman, Ravelina R. Velasco, Ma. Jodecel C. Danting, John A. H. Benzie

**Affiliations:** ^1^WorldFish, Penang, Malaysia; ^2^Department of Aquatic Resources, Ecology and Management, College of Fisheries, Central Luzon State University, Muñoz, Philippines; ^3^Freshwater Aquaculture Center, Central Luzon State University, Muñoz, Philippines; ^4^Bureau of Fisheries and Aquatic Resources, National Freshwater Fisheries Technology Center, Central Luzon State University Compound, Science City of Munoz, Philippines; ^5^School of Biological Earth and Environmental Sciences, University College Cork, Cork, Ireland

**Keywords:** aquaculture, nile tilapia (*Oreochromis niloticus*), genetic improvement, single nucleotide polymorphism (SNP), discriminant analysis of principal components (DAPC), strain identification

## Abstract

Nile tilapia (*Oreochromis niloticus*) is among the most important finfish in aquaculture, particularly in Asia. Numerous genetically improved strains of Nile tilapia have been developed and disseminated through formal and informal channels to hatcheries, many of which operate at a relatively small scale in developing countries. The primary objective of this study was to assess the extent to which molecular genetic tools can identify different and interrelated strains of Nile tilapia in Bangladesh and the Philippines, two globally significant producers. A tool was developed using a low-density panel of single-nucleotide polymorphisms (SNPs), genotyping-by-sequencing and discriminant analysis of principal components (DAPC). When applied to 2,057 samples from 205 hatcheries in Bangladesh and the Philippines, for hatcheries where the hatchery-identified strain was one of the sampled core populations used to develop the tool, hatchery-identified and DAPC-assigned hatchery-level strains were in agreement in 74.1% of cases in Bangladesh and 80.6% of cases in the Philippines. The dominant hatchery-identified and DAPC-assigned strains were GIFT, in Bangladesh, and GET-ExCEL—a composite strain partially derived from GIFT—in the Philippines.

## Introduction

By weight, Nile tilapia (*Oreochromis niloticus*) is the most important finfish species in global aquaculture after carp (*Cyprinidae*) (Cai et al., 2019). Nile tilapia production has increased substantially over the past 20 years aided by the widespread adoption of technologies to produce monosex all-male tilapia and the development of genetically improved strains (Gupta and Acosta, 2004; ADB, 2005; Ponzoni et al., 2010). Genetic improvement of Nile tilapia was initiated in 1988 with the creation of the “Genetically Improved Farmed Tilapia” (GIFT) strain, initially in the Philippines by ICLARM (now WorldFish) and its partners (Gupta and Acosta, 2004). The GIFT strain has subsequently been disseminated widely in Asia and has genetically contributed to numerous “GIFT-derived” strains—including “Genetically Enhanced Tilapia—Excellent strain that has Comparable advantage over other tilapia strains for Entrepreneurial Livelihood projects” (GET-ExCEL), “Brackishwater Enhanced Selected Tilapia” (BEST), and Molobicus in the Philippines. Genetic improvement programs based on populations wholly descended from the original GIFT population are maintained by WorldFish (GIFT-WF, Malaysia), the Bureau of Fisheries and Aquatic Resources National Freshwater Fisheries Technology Center and its partners (GIFT FeedMix Fortified; GIFTFF, Philippines), and GenoMar (GenoMar Supreme Tilapia, Philippines), among others (Gupta and Acosta, 2004; Eknath and Hulata, 2009; Ponzoni et al., 2010; Ordoñez et al., 2014, 2017). In southeast Asia, other strains, such as Chitralada (Thailand) and “Freshwater Aquaculture Center Selected Tilapia” (FaST, Philippines), have been developed independently of GIFT (Pullin, 1988; Ordoñez et al., 2017).

In Bangladesh, production of Nile tilapia was 380,000 metric tons in 2017–2018, making it the fourth largest tilapia producer globally (DOF, 2018). Nile tilapia is also the second most important farmed fish species after milkfish (*Chanos chanos*) in the Philippines, with 267,700 metric tons produced in 2017 (Bersales and Bautista, 2018), concentrated in the Central Luzon and Calabarzon regions. Numerous genetically improved Nile tilapia strains are now available in these countries. However, the origins, genetic purity, and level of genetic improvement of strains supplied by hatcheries are not always known.

Genetically improved strains of tilapia known to have been introduced into Bangladesh include Chitralada from Thailand (1974, 1987, 2002, 2010), GIFT from the Philippines (1994, 1996), GenoMar Supreme Tilapia (GST) from the Philippines (2003), GIFT-WF from Malaysia (2005, 2012), GIFU from China (2008), and FaST from the Philippines (2011) (Ponzoni et al., 2010; Hussain et al., 2014). Other undocumented introductions of improved strains have likely occurred. In the Philippines, numerous locally developed strains have been developed. In addition, GIFT-WF from Malaysia was introduced for strain comparison purposes in 2012 (Battad, 2013) and for direct dissemination to farmers in 2014–2015 (Worldfish, 2015).

Lack of clarity concerning seed origins and distribution makes it difficult to understand the level of adoption and performance of fish strains in farming systems. Corresponding assessment of returns on investment may be inaccurate, and decisions on future actions by policy makers and investors are adversely affected. Numerous tools relying on a small number of genetic markers to identify genetically homogenous inbred crop lines and clonal horticultural varieties have been developed, and the extent of seed misidentification in crops is increasingly recognized in agriculture through the application of these methods (Rabbi et al., 2015; Chen et al., 2016; Floro et al., 2018; Kosmowski et al., 2019; Wineman et al., 2020). However, the development of comparable tools to distinguish between strains of outcrossing aquaculture species is challenging, and information on the extent of misidentification of tilapia strains is currently limited (Baggio et al., 2016; Oponda et al., 2017; Ordoñez et al., 2017; Moses et al., 2020).

The broad objective of the present study was to assess the extent to which molecular genetic tools can identify different and interrelated strains of tilapia and then test, to the extent possible, the actual prevalence of different strains in commercial hatcheries in Bangladesh and the Philippines. The specific aims were to (i) identify single-nucleotide polymorphisms (SNPs) for Nile tilapia, (ii) examine SNP genetic affinities among “core breeding populations” of widely disseminated genetically improved strains, (iii) identify a subset of SNPs that allows core breeding populations of Nile tilapia to be distinguished, and (iv) validate hatchery-identified strains.

## Materials and Methods

### Sampling Core Breeding Populations and Hatcheries

**Figure 1:**
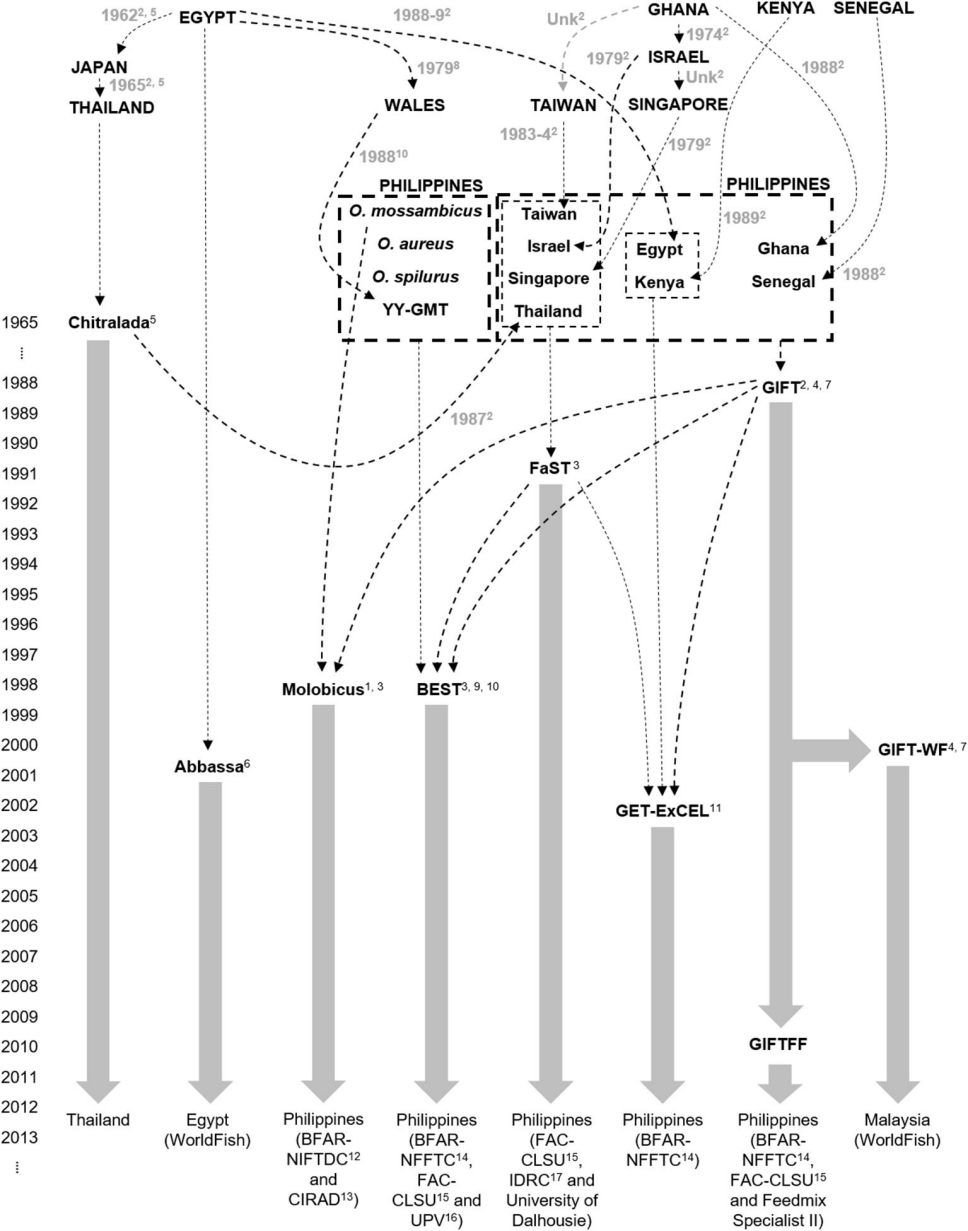
Genetic origins of *Oreochromis niloticus* in sampled core populations: ^1^Bartie et al. (2020), ^2^Eknath et al. (1993), ^3^Ordoñez et al. (2014), ^4^Ponzoni et al. (2010), ^5^Pullin (1988), ^6^Rezk et al. (2002), ^7^Rodriguez (2006), ^8^Scott et al. (1989), ^9^Tayamen et al. (2002), ^10^Tayamen et al. (2004), ^11^Tayamen (2004), ^12^Bureau of Fisheries and Aquatic Resources National Integrated Fisheries Technology Development Center, ^13^Center de Cooperation Internationale en Recherche Agronomique pour le Development, ^14^Bureau of Fisheries and Aquatic Resources National Freshwater Fisheries Technology Center, ^15^Freshwater Aquaculture Center Central Luzon State University, ^16^University of the Philippines Visayas, ^17^International Development Research Center.

**Figure 2:**
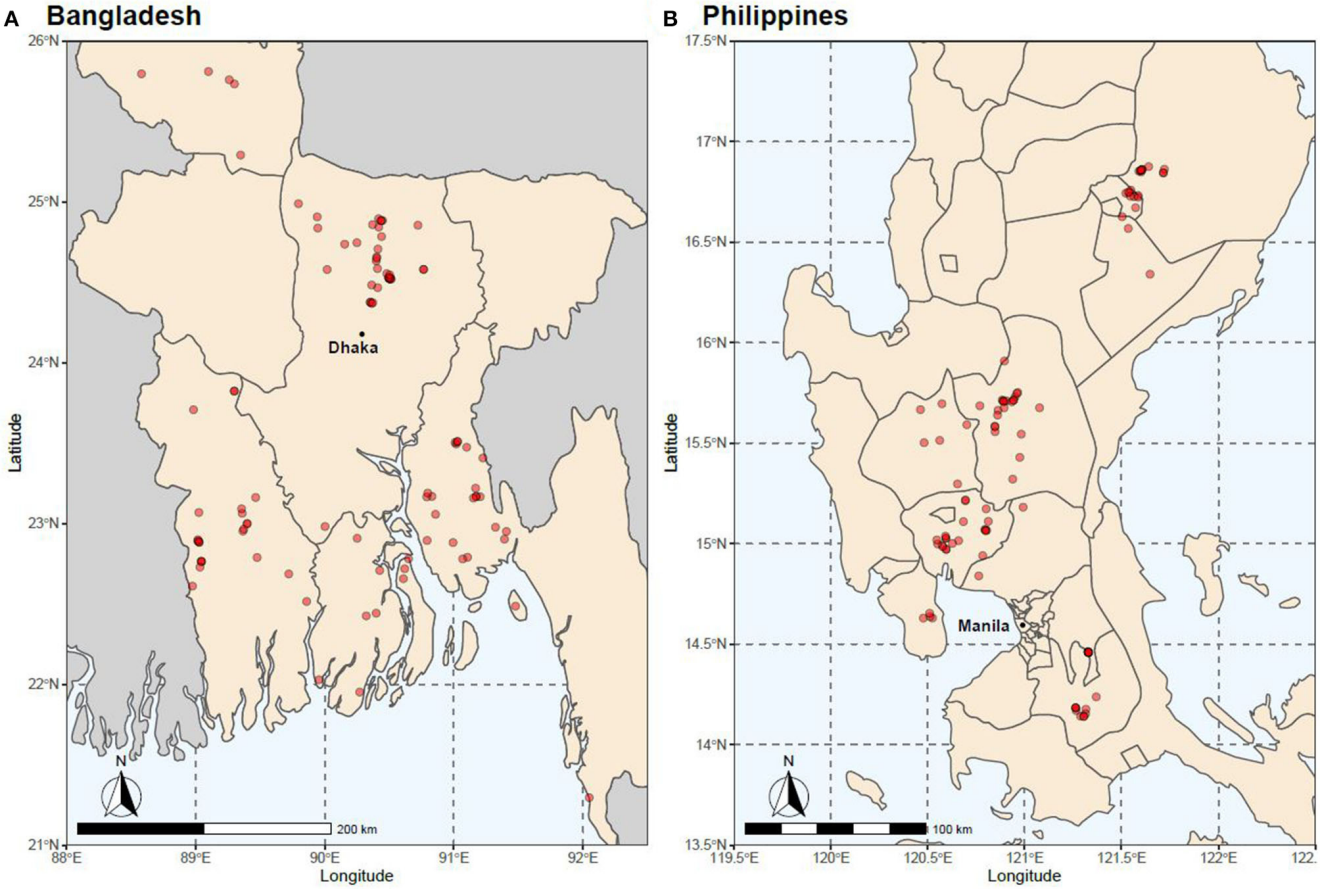
Location of hatcheries from which samples were obtained in **(A)** Bangladesh and **(B)** the Philippines.

In 2015, a total of 852 fin-clip samples were obtained from 10 core breeding populations—two “GIFT,” four “GIFT-derived,” and four “non-GIFT” strains available in Bangladesh and/or the Philippines (Table 1) whose complex relationships are summarized in Figure 1. The number of individuals sampled per strain ranged from 21 to 122. Separately, a total of 2,057 fin clip samples of broodstock were obtained from tilapia hatcheries in Bangladesh and the Philippines. Sampled hatcheries provided details of the origins of their broodstock, herein referred to as the “hatchery-identified strain.” With the exception of GIFU (one hatchery in Bangladesh) and GenoMar (two hatcheries in Bangladesh and one in the Philippines), all hatchery-identified strains are represented in Figure 1. The GIFU strain was developed in China, but beyond that, its origins are unclear in the literature (Hasan et al., 2014). The GenoMar strain is wholly descended from the original GIFT population (Rodriguez, 2006; Ponzoni et al., 2010). In total, 1,053 samples were obtained from fish held by 106 private and public hatcheries in Bangladesh (Figure 2A), and 1,004 samples were obtained from 99 private hatcheries in the Philippines (Figure 2B). All fish sampled for this study were handled and biopsied using standard practices routinely employed in commercial tilapia operations. Fish were fin clipped using non-lethal, humane methods in accordance with the Guiding Principles of the Animal Care, Welfare and Ethics Policy of the WorldFish Center (Worldfish, 2004).

**Table 1:** Sampled genetically improved farmed tilapia (GIFT), GIFT-derived and non-GIFT core breeding populations.

**Strain**	**Group**	**Location**	**Organization**	**Count**	**Species**
GIFT-WF	GIFT	Malaysia	WorldFish	99	*O. niloticus*
GIFTFF	GIFT	Philippines	BFAR NFFTC^a^	47	*O. niloticus*
BEST	GIFT-derived	Philippines	BFAR NFFTC^a^	47	Hybrid^e^
GET-ExCEL	GIFT-derived	Philippines	BFAR NFFTC^a^	94	*O. niloticus*
Molobicus	GIFT-derived	Philippines	BFAR NIFTDC^b^	172	Hybrid^f^
Nile × Moss	GIFT-derived	Philippines	BFAR NIFTDC^b^	21	Hybrid^g^
Abbassa	Non-GIFT	Egypt	WorldFish	122	*O. niloticus*
Chitralada	Non-GIFT	Thailand	AIT^c^	94	*O. niloticus*
FaST	Non-GIFT	Philippines	FAC CLSU^d^	120	*O. niloticus*
*O. mossambicus*	Non-GIFT	Philippines	BFAR NIFTDC^b^	36	*O. mossambicus*
Total				852	

a *Bureau of Fisheries and Aquatic Resources, National Freshwater Fisheries Technology Center*;

b *National Integrated Fisheries Technology Development Center*;

c *Asian Institute of Technology*;

d *Freshwater Aquaculture Center, Central Luzon State University*;

e *advanced-generation hybrid of O. aureus, O. niloticus, O. mossambicus, and O. spilurus (Ordoñez et al., 2014)*;

f *advanced-generation hybrid of O. niloticus and O. mossambicus (Ordoñez et al., 2014)*;

g *first filial (F1) generation hybrid of O. niloticus and O. mossambicus*.

### Sequencing and SNP Discovery

A total of 852 samples from core breeding populations were genotyped by Diversity Arrays Technology (DArT), using the DArTseq genotyping-by-sequencing platform, according to the methods detailed in Lind et al. (2017). DNA was extracted by DArT using a Macherey–Nagel (Düren, Germany) NucleoMag 96 Tissue Kit and a NucleoMag SEP Magnetic Separator 744,900 to allow automated separation of high-quality DNA on a Tecan (Männedorf, Switzerland) Freedom Evo robotic liquid handler. Samples were genotyped along with 35 duplicates and 517 samples from African populations. In total, 21,195 SNP loci were identified (Supplementary Material 1). Prior to analyses, duplicate and African samples not relevant to the current study were excluded, and quality control procedures were implemented—SNPs with a minor allele frequency of <0.01 (1852 SNPs), and those for which more than 25% of individuals had missing genotypes (an additional 5678 SNPs), were excluded. This is herein referred to as the “full DArTseq panel.” The removal of African samples prior to quality control inflated the number of excluded SNPs—as SNPs unique to, or disproportionately expressed in, African populations were removed. All analyses of SNP data were conducted using R (R Core Team, 2018).

To assess the ability to correctly identify the strain of individuals sampled from the core breeding populations using the full DArTseq panel, a 4-fold cross-validation approach was adopted by (i) masking the strain of 25% of animals, selected at random, from within each core breeding population, (ii) performing DAPC (Jombart et al., 2010) using the full DArTseq panel and the unmasked training set of individuals, (iii) inferring the strain of masked individuals, and (iv) determining the proportion of masked individuals that were correctly assigned to their strain. The DAPC analyses were performed using the *dapc* function of the *adegenet* package (Jombart and Ahmed, 2011). *dapc* default settings were adopted except that *n.da* and *n.pca* were both set to the number of principal components achieving the lowest root mean squared error outputted by the *xvalDapc* function—which implements a stratified cross-validation of DAPC using varying numbers of principal components, while keeping the number of discriminant functions fixed (Di Prinzio et al., 2015; Jombart and Collins, 2015). *xvalDapc* default settings were also adopted except that *training.set* was set to 0.75, *result* was set to “groupMean” and *n.rep* was set to 100. Strain predictions were then made using the DAPC results and the *predict.dapc* function (default settings). Masked individuals were determined to be correctly assigned if the core breeding population from which the individual was sampled had the greatest posterior membership probability. This procedure was repeated 10 times to reduce bias due to sample allocation to the training datasets.

To investigate genetic affinities among the 10 core populations, unsupervised k-means clustering was undertaken using the *glPca, find.clusters*, and *dapc* functions of the *adegenet* package (Version 2.1.1 Jombart and Ahmed, 2011; Jombart and Collins, 2015). The *glPca* function was used to undertake principal component analyses (PCA), using default settings with *nf* set to 500—to ensure that data for all pertinent principal components were retained. The *find.clusters* function was then used to identify the number of groups that usefully describe the data, by plotting the Bayesian information criterion (BIC) for increasing values of k (Jombart and Collins, 2015). Default settings of *find.clusters* were adopted but with *n.start* set to 1,000—to ensure convergence of the K-means algorithm—*n.pca* set to 500—to include all retained principal components—and max.n.clust set to 40—to evaluate levels of k from 1 to 40. Discriminant analysis of principal components (DAPC, Jombart et al., 2010) was then performed for values of *k* increasing from 2 to 15 using the *dapc* function of *adegenet*. Default settings were adopted except that the number of principal components retained (*n.pca*) was determined by the *optim.a.score* function—to avoid overfitting (Di Prinzio et al., 2015)—and n.da was set to 100—so that all discriminant axes were retained.

To reduce costs, simplify implementation, and ease the computational burden of strain identification for the hatchery samples, a subset of informative SNPs was identified. This was achieved by computing the pairwise ***F***_***ST***_ and **δ** values for each SNP across all possible pairwise combinations of the 10 core breeding populations (45 combinations). ***F***_***ST***_ values were computed as **(*H***_***T***_**−*H***_***S***_**)/*H***_***T***_, where ***H***_***T***_ is the expected heterozygosity across the total population, and ***H***_***S***_ is the expected heterozygosity of the of the individual core breeding populations (Weir and Cockerham, 1996), and **δ** was computed as |***p***_***Ai***_**−*p***_***Aj***_|, where ***p***_***Ai***_ and ***p***_***Aj***_ are the frequencies of allele A in the *i*th and *j*th core breeding populations, respectively (Supplementary Material 2). A cutoff criteria to include the top 75 ranked SNPs for every pairwise combination provided a subset of 1,297 unique SNPs using ***F***_***ST***_ and 1,214 unique SNPs using **δ**. When these lists of SNPs were combined, 1,387 unique SNPs that met the quality control criteria were identified, and these were defined as the “full list of informative SNPs.”

### Hatchery Samples

Hatchery samples were genotyped for the full list of informative SNPs using DArTcap, a low-cost targeted genotyping method that applies a selective step after complexity reduction to genotype-specific markers from DArTseq representations (Chen et al., 2016). With DArTcap, 1,334 SNPs were expressed. Quality control on DArTcap data was undertaken—SNPs with a minor allele frequency of <0.01 (85 SNPs), and those for which more than 25% of individuals had missing genotypes (an additional 10 SNPs), were excluded. Of the remaining SNPs, 789 corresponded to those from DArTseq (707 were in the full list of informative SNPs) and were used to assign hatchery samples to “DAPC-assigned strains.” These 789 SNP were referred to as the “reduced subset of informative SNP.” Using core population data, the 4 fold cross validation scheme adopted for the full DArTseq panel, detailed above, was repeated for the reduced subset of informative SNPs. DAPC strain assignment was then undertaken for hatchery samples with the *predict.dapc* function—after completing DAPC using core population data—by assigning individuals to the strain with the greatest posterior membership probability (referred to as “individual-fish level” assignment). Hatcheries were then assigned the strain represented by the most individuals (i.e., the “modal strain,” referred to as “hatchery-level” assignment). In addition, DAPC assignment to “groups” was undertaken by allocating core populations to ancestral groups (Table 1)—GIFT, GIFT-derived, non-GIFT (*O. niloticus*), and non-GIFT (*O. mossambicus*)—and repeating the assignment process.

## Results

### Core Breeding Populations

**Figure 3:**
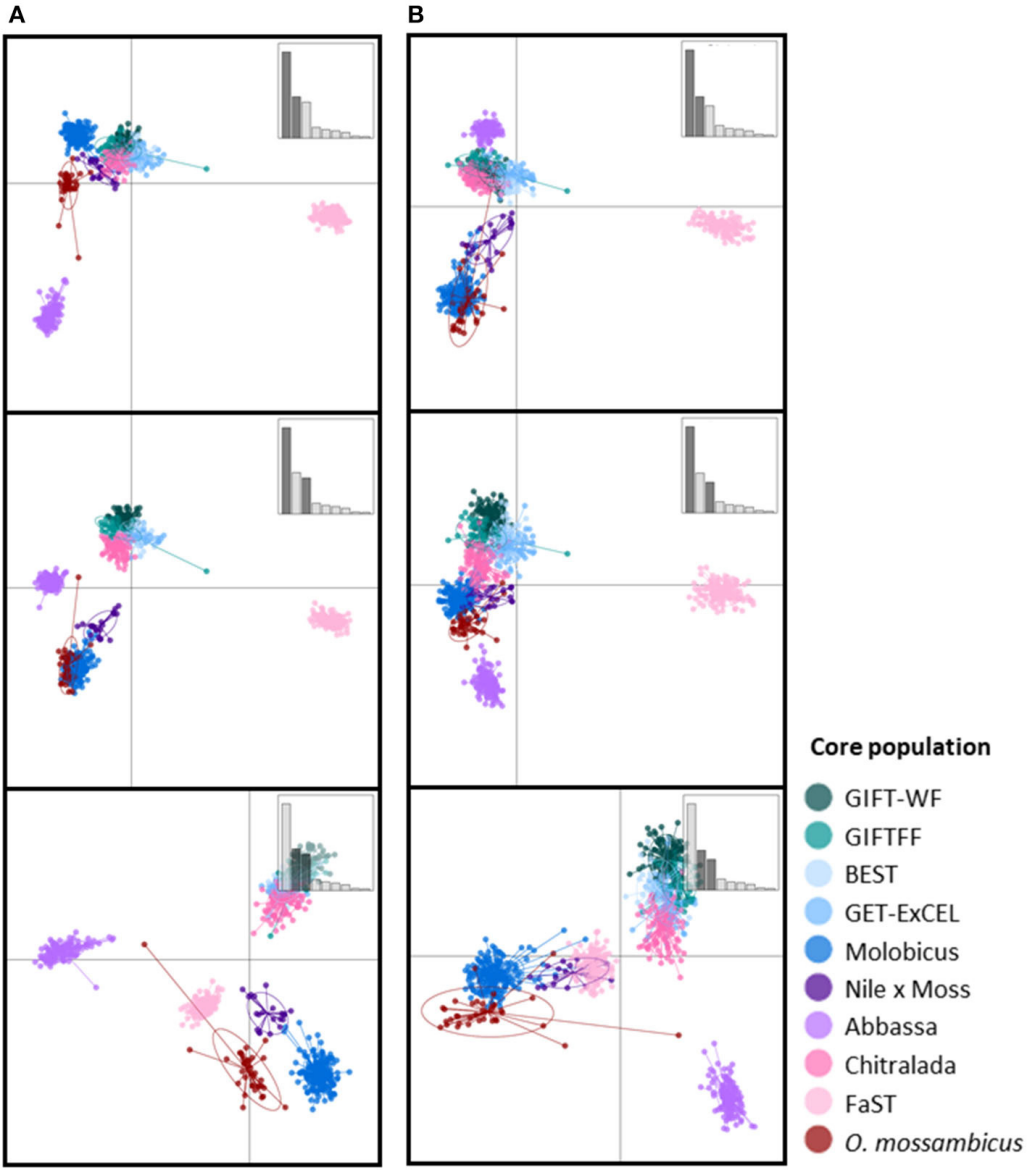
Scatterplots of the first three discriminant functions from discriminant analysis of principal components (DAPC) using **(A)** the full DArTseq panel and **(B)** the reduced subset of informative SNPs. Insets show bar plots of discriminant analysis eigenvalues, and those axes used in a given plot are darkened (so top to bottom axes 1–2, 1–3, 2–3).

The scatterplots of the first two DAPC discriminant functions using the full DArTseq panel (Figure 3A) revealed three distinct clusters of non-hybrid *O. niloticus* strains—Abbassa and FaST formed two distinct clusters, whereas there was substantial overlap among Chitralada, ExCEL, GIFT-WF, and GIFTFF strains. Individuals from the BEST strain, despite descending from multiple tilapia species (Figure 1) also clustered with Chitralada, ExCEL, GIFT-WF, and GIFTFF. Furthermore, the three strains descended from *O. mossambicus* formed non-overlapping clusters in plots involving the third discriminant function. Using the reduced subset of informative SNP, these four clusters—i) Abbassa, ii) FAST, iii) GIFT-WF, GIFTFF, Chitralada, and GET-ExCEL, and iv) Molobicus, Nile × Moss, and *O. mossambicus*—were evident in the plot of the first two DAPC discriminant functions.

**Figure 4:**
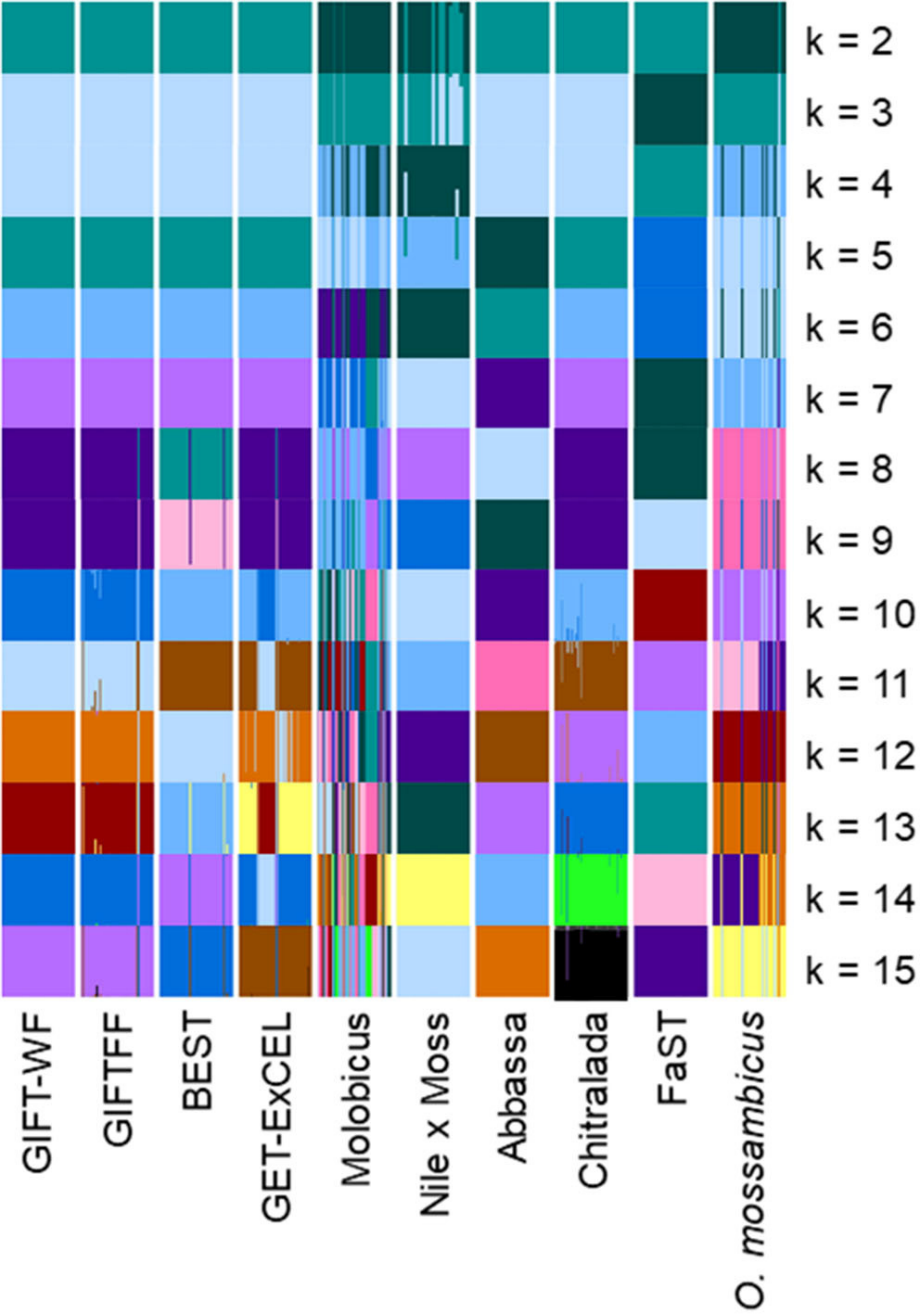
Unsupervised k-means clustering of individuals in core breeding populations performed using discriminant analysis of principal components (DAPC) for differing numbers of groups (*k*) using the full DArTseq panel. Each of the groups is represented by a different color. Vertical lines represent the cluster membership probability of individuals.

Plots of the Bayesian information criterion (BIC) for increasing values of k (Jombart and Collins, 2015) did not reveal an optimal number of clusters to usefully describe the data but indicated that no more than 15 groups would be appropriate (Supplementary Material 3). Using the full DArTseq panel, when two groups (*k* = 2) were defined using unsupervised k-means clustering (Figure 4), individuals from the non-hybrid *O. niloticus* strains and BEST clustered together in one group and hybrid and non-hybrid *O. mossambicus* strains in the other. When three groups were defined (*k* = 3), FaST formed its own group, and when five groups were defined (*k* = 5) Abbassa formed its own group. Notably, most individuals from the GIFT-WF and GIFTFF strains were assigned to a single group, even when 15 groups (*k* = 15) were defined, reflecting the shared origins of these strains (Figure 1 and Table 1). In contrast, Molobicus individuals were assigned to multiple groups, indicating substantial heterogeneity among individuals within the population.

**Figure 5:**
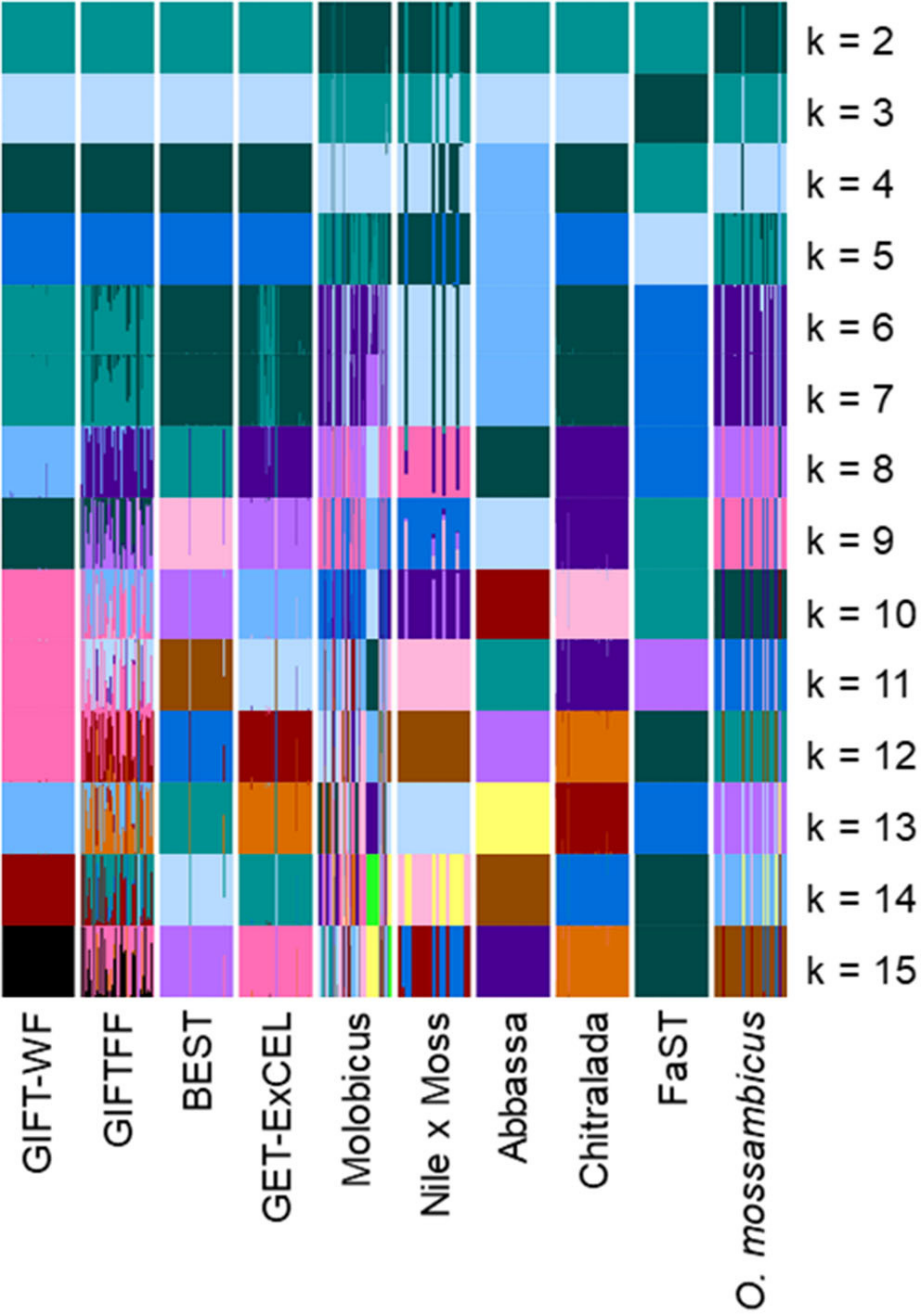
Unsupervised k-means clustering of individuals in core breeding populations performed using discriminant analysis of principal components (DAPC) for differing numbers of groups (*k*) using the reduced subset of informative SNPs. Each of the groups is represented by a different color. Vertical lines represent the cluster membership probability of individuals.

The application of unsupervised k-means clustering to individuals from core breeding populations using the reduced subset of informative SNP (Figure 5) resulted in more distinct partitioning of *O. niloticus* strains—reflecting intentional ascertainment bias in the selection of informative SNP toward those under selection or affected by genetic drift (Bradbury et al., 2011; Grewe et al., 2015; Gilbey et al., 2016). However, using the reduced subset of informative SNP, individuals from GIFTFF did not form a distinct cluster, clustering primarily with individuals from GIFT-WF or GET-ExCEL. Individuals from the Molobicus core population also clustered with individuals from multiple other core populations.

Prediction efficiency of DAPC for core breeding populations using the full DArTseq panel was high for all strains, with >93.3% correct assignment (Table 2A). GIFTFF had the lowest prediction efficiency, but the majority of incorrectly assigned individuals in this case (4.2% of 6.7%) were assigned to GIFT-WF which, like GIFTFF, is descended from the original GIFT population (Figure 1). The most notable difference between the prediction efficiency of the full DArTseq panel and the reduced subset of informative SNP (Table 2B) was in the hybrid (Nile × Moss), in which, in the case of the reduced subset of informative SNP, prediction efficiencies were substantially lower, due to the erroneous assignment of 10.0% of individuals to the Molobicus strain. In addition, 5.6% of *O. mossambicus* individuals were assigned to the Abbassa strain using the reduced subset of informative SNP. It is unclear why these strains were more affected than others by the adoption of the reduced subset of informative SNP, but it is notable that these stains had the lowest number of samples from core populations (21 and 36, respectively).

**Table 2:** Prediction efficiency expressed as a percentage from discriminant analysis of principal components (DAPC) of core breeding populations using **(A)** the full DArTseq panel and **(B)** the reduced subset of informative single-nucleotide polymorphisms (SNPs).

	**GIFT**		**GIFT-derived**				**Non-GIFT**			
	**GIFT-WF**	**GIFTFF**	**BEST**	**GET-ExCEL**	**Molobicus**	**Nile × Moss**	**Abbassa**	**Chitralada**	**FaST**	***O. mossambicus***
**A**										
GIFT-WF	**99.6**	0.4								
GIFTFF	4.2	**93.3**		2.5						
BEST			**95.0**	5						
GET-ExCEL		1.7	0.8	**97.5**						
Molobicus		0.5		0.2	**97.4**	0.5		1.4		
Nile × Moss						**100**				
Abbassa							**100**			
Chitralada								**100**		
FaST									**100**	
*O. mossambicus*							1.1			**98.9**
**B**										
GIFT-WF	**100**									
GIFTFF	4.2	**89.2**	1.7	4.2				0.8		
BEST			**98.3**	1.7						
GET-ExCEL			1.3	**98.8**						
Molobicus				0.9	**97.2**	0.2		1.2		0.5
Nile × Moss					10.0	**86.0**		4.0		
Abbassa							**100**			
Chitralada		1.3						**98.8**		
FaST									**100**	
*O. mossambicus*					1.1	2.2	5.6			**91.1**

*True populations are in rows, and inferred populations are in columns. The diagonal (in bold) contains the percentage of correct assignments across 10 repetitions of a fourfold cross validation. Off-diagonals contain the percentage of erroneous allocations*.

### Hatchery Populations

**Figure 6:**
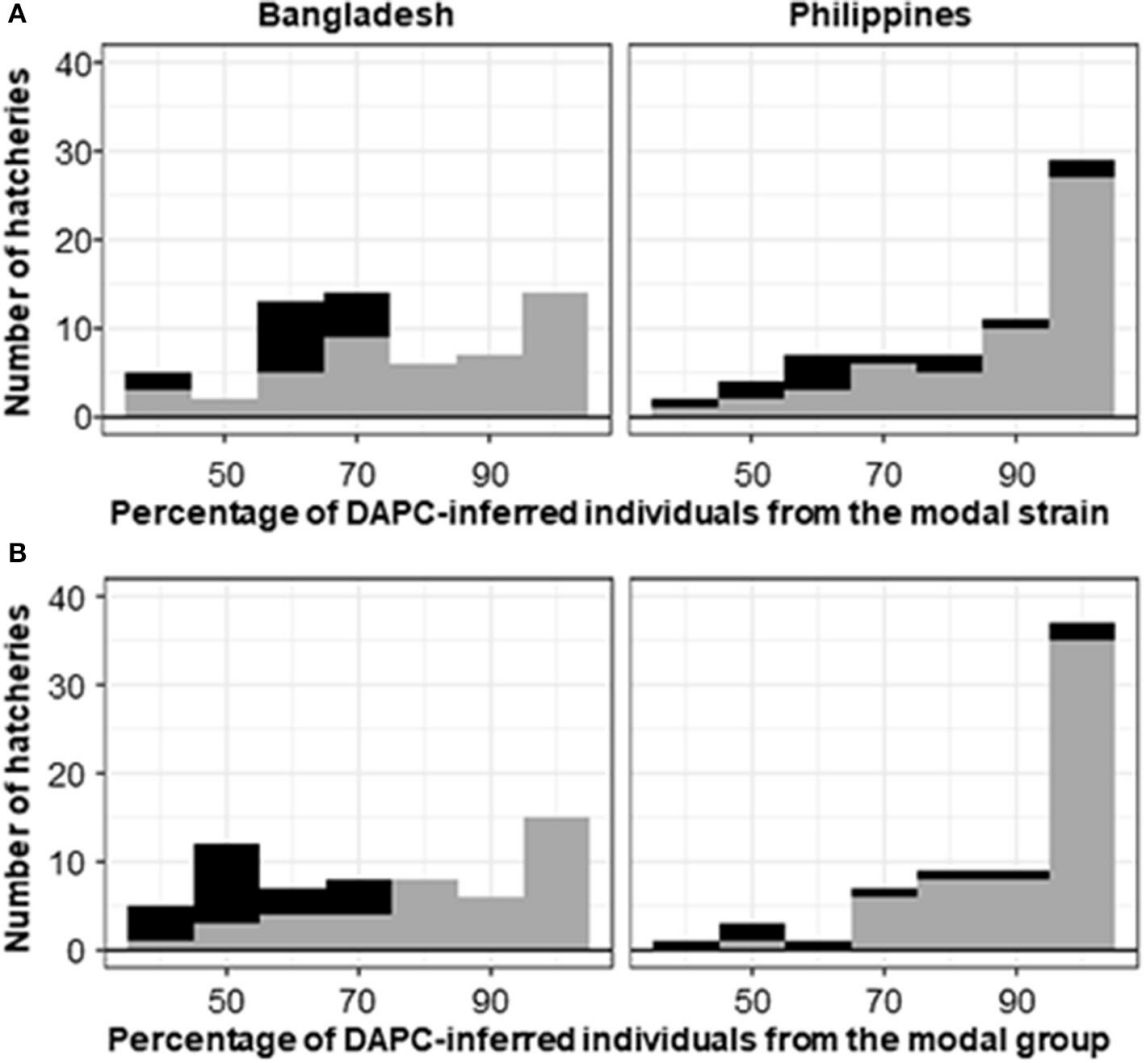
Histogram of the percentage of DAPC-assigned individuals from a hatchery in the modal DAPC-assigned **(A)** strain or **(B)** group. Gray shading indicates hatcheries where the hatchery-identified strain or group matched the DAPC-assigned (i.e., modal) strain or group. Black shading indicates mismatches. Hatcheries for which the hatchery-identified strain was unknown (45 from Bangladesh and 32 from the Philippines) are excluded.

Numerous mismatches between the hatchery-identified strain and the DAPC-inferred strain were evident. This was most apparent at the individual-fish level. In Bangladesh, where the hatchery-identified strain was one of the sampled core populations (i.e., GIFT, Chitralada, or FaST), hatchery-identified and DAPC-inferred strains were in agreement in 67.3% of cases (Table 3A). In comparison, at the hatchery level, hatchery-identified and DAPC-inferred strains were in agreement in 74.1% of cases (Table 3B; see also Figure 6A). At the hatchery level, the most common hatchery-identified strain was GIFT (47% hatcheries), of which 50.0% were assigned to GIFT-WF and 26.0% to GIFTFF using DAPC. Of the 42% of Bangladeshi hatcheries with an unknown strain, 51.1% were assigned to the Chitralada strain and 44.5% to GIFT-WF or GIFTFF. Overall, 32.1% of Bangladeshi hatcheries were assigned to Chitralada, 26.4% to GIFT-WF, and 33.0% to GIFTFF.

**Table 3:** Degree of agreement between hatchery-identified strain and DAPC-assigned strain expressed as a percentage.

		**DAPC-assigned strain**										
		**GIFT**		**GIFT-derived**				**Non-GIFT**					
**Country**	**Hatchery-identified strain**	**GIFT-WF**	**GIFTFF**	**BEST**	**GET-ExCEL**	**Molobicus**	**Nile × Moss**	**Abbassa**	**Chitralada**	**FaST**	***O. mossambicus***	**Unassigned**	**N (individuals)**
**A**													
Bangladesh	GenoMar	**10.0**	**55.0**						35.0				20
	GIFT	**41.4**	**27.1**		7.8	1.6			21.3		0.2	0.6	498
	GIFU	10.0	60.0		20.0				10				10
	Chitralada		35.0						**65.0**				20
	FaST	10.0	13.3		5.0				13.3	**58.3**			60
	Unknown	9.4	34.2		10.1	0.9	0.2		44.7			0.4	445
	Total	24.4	30.3		8.5	1.1	0.1		31.7	3.3	0.1	0.5	1,053
Philippines	GenoMar									100			10
	GIFT	**10.0**	**50.0**						40.0				20
	BEST		25	**40.0**	30.0				5.0				20
	GET-ExCEL	0.8	4.5	4.8	**79.9**				7.7	2.1		0.3	378
	Chitralada	5.0			65.0				**20.0**	10.0			20
	FaST	0.4	4.3	0.4	20.5				6.4	**67.9**			234
	Unknown		9.9	4.7	40.1			0.3	32	12.7		0.3	322
	Total	0.7	7.4	4.2	49.6			0.1	15.9	21.9		0.2	1,004
**B**													
Bangladesh	GenoMar		**100**										2
	GIFT	**50.0**	**26.0**		4.0	2.0			18.0				50
	GIFU		100										1
	Chitralada		50.0						**50.0**				2
	FaST		16.7						16.7	**66.7**			6
	Unknown	6.7	37.8		4.4				51.1				45
	Total	26.4	33.0		3.8	0.9			32.1	3.8			106
Philippines	GenoMar									100			1
	GIFT		**50.0**						50.0				2
	BEST			**50.0**	50.0								2
	GET-ExCEL				**94.6**				2.7	2.7			37
	Chitralada				100								1
	FaST		4.2		16.7				8.3	**70.8**			24
	Unknown		9.4	3.1	40.6				31.3	15.6			32
	Total		5.1	2.0	54.5				14.1	24.2			99

*Hatchery-identified strains are in rows and inferred populations are in columns. Individuals were assigned to the strain with the greatest posterior membership probability. Data are presented at **(A)** the individual-fish level and **(B)** at the hatchery level with DAPC-assigned strain being the strain represented by the most individuals (i.e., the “modal strain”). Numbers in bold represent the percentage of individuals/hatcheries for which the hatchery-identified strain and DAPC-assigned strain were the same*.

For the Philippines, where the hatchery-identified strain was one of the sampled core populations (i.e., GIFT, BEST, GET-ExCEL, Chitralada, or FaST), hatchery-identified and DAPC-inferred strains were in agreement in 72.1% of cases at the individual-fish level (Table 3A) and 80.6% of cases at the hatchery level (Table 3B; see also Figure 6A). At the hatchery level, the dominant hatchery-identified strains were GET-ExCEL−37% (of which 94.6% were assigned to GET-ExCEL using DAPC) and FaST−24% (of which 70.8% were assigned to FaST using DAPC). Of all the sampled hatcheries in the Philippines, 14.1% were assigned to Chitralada, 54.5% to GET-ExCEL, 24.2% to FaST, and 5.1% to GIFTFF using DAPC (Table 3B).

Since many of the strains were closely related, it was reasoned that some mismatches between hatchery-identified strain and DAPC-inferred strain were potentially due to DAPC misassignment among closely related strains. Accordingly, the analysis was repeated to assign individuals and hatcheries to one of the groups of GIFT, GIFT-derived, non-GIFT *O. niloticus* and *O. mossambicus* (Table 4). However, only minor differences in the concordance between hatchery-identified and DAPC-inferred results were evident when data were analyzed as groups, rather than strains. At the individual-fish level, where the hatchery-identified strain was one of GIFT, BEST, GET-ExCEL, Chitralada, or FaST, hatchery-identified and DAPC-inferred groups were in agreement in 66.1 and 82.6% of cases, for Bangladesh and the Philippines, respectively (Table 4A). At the hatchery level, hatchery-identified and DAPC-inferred groups were in agreement in 69.0 and 87.9% of cases, respectively (Table 4B; see also Figure 6B). GIFT was the most prevalent DAPC-assigned group in Bangladesh (50.0%), and GIFT-derived strains (48.5%) were most dominant in the Philippines. The non-GIFT *O. niloticus* DAPC-assigned group represented similar percentages in both Bangladesh (42.5%) and the Philippines (46.5%).

**Table 4:** Degree of agreement between hatchery-identified strain and DAPC-assigned group expressed as a percentage, where group is defined as GIFT, GIFT-derived, or non-GIFT *O. niloticus* or *O. mossambicus*.

		**DAPC-assigned group**					
**Country**	**Hatchery-identified strain**	**GIFT**	**GIFT-derived**	***Non-GIFT O. niloticus***	***O. mossambicus***	**Unassigned**	**N (individuals)**
**A**							
Bangladesh	GenoMar	**45.0**	10.0	45.0			20
	GIFT	**64.7**	11.6	23.1	0.2	0.4	498
	GIFU	60.0	30.0	10.0			10
	Chitralada	20.0	5.0	**75.0**			20
	FaST	23.3	1.7	**75.0**			60
	Unknown	35.7	17.3	46.3		0.7	445
	Total	48.8	13.5	37.1	0.1	0.5	1053
Philippines	GenoMar			100			10
	GIFT	**65.0**		35.0			20
	BEST	5	**90.0**	5			20
	GET-ExCEL	3.4	**84.9**	11.6			378
	Chitralada	5.0	15.0	**80.0**			20
	FaST	3.0	15.8	**79.9**		1.3	234
	Unknown	10.6	41.6	47.8		0	322
	Total	6.9	51.1	41.7		0.3	1,004
**B**							
Bangladesh	GenoMar	**50.0**		50.0			2
	GIFT	**66.0**	10	24.0			50
	GIFU	100					1
	Chitralada			**100**			2
	FaST	16.7		**83.3**			6
	Unknown	37.8	6.7	55.6			45
	Total	50.0	7.5	42.5			106
Philippines	GenoMar			100			1
	GIFT	**50.0**		50.0			2
	BEST		**100**				2
	GET-ExCEL		**89.2**	10.8			37
	Chitralada			**100**			1
	FaST	4.27	8.33	**87.5**			24
	Unknown	9.3	34.3	56.3			32
	Total	5.2	48.5	46.5			99

*Data are presented at **(A)** the individual-fish level, **(B)** at the hatchery level with DAPC-assigned group being the group represented by the most individuals (i.e., the “modal group”). Numbers in bold represent the percentage of individuals/hatcheries for which the hatchery-identified strain and DAPC-assigned strain were the same*.

Varying levels of DAPC assignment consistency among sampled individuals within hatcheries were evident, with 100% of individuals assigned to the same DAPC-inferred strain or group in some hatcheries but only 40% being assigned to a common DAPC-inferred strain (Figure 6A) or group (Figure 6B) in others. For hatcheries where the hatchery-identified and DAPC-assigned strain or group did not match, it was not possible to independently verify which was correct. However, where 75% or more of DAPC-assigned individuals from a hatchery were in the modal DAPC-assigned strain (or group), concordance between hatchery-identified and DAPC-inferred strain (or group) was strong—indicating that our SNP and assignment method was accurate for hatcheries in which this threshold was met (Figure 6). The percentage of DAPC-assigned individuals from the modal DAPC-assigned strain (or group) is a simple measure of confidence in assignment. At the strain level, 74 hatcheries (27 in Bangladesh and 47 in the Philippines) were above this 75% “confidence threshold” of which 69 exhibited a match between hatchery-identified strain and DAPC-inferred strain (Figure 6A). At the group level, 85 hatcheries (29 in Bangladesh and 56 in the Philippines) were above the threshold, of which 81 matched. The high proportion of hatcheries with 100% of individuals assigned to the same DAPC-inferred strain (or group) in the Philippines was likely due to the high proportion of hatcheries maintaining the FaST and GET-ExCEL strains. The FaST strain was most easily distinguished from other strains using our subset of informative SNPs and assignment method (Figures 3–5), and a high degree of concordance between hatchery-identified strain and DAPC assigned strain was evident in the case of GET-ExCEL (Table 3).

## Discussion

Sampled core populations were in many cases interrelated and descended in part, or full, from common founder populations (Figure 1) and, given their known parent selection and mating strategies, are likely to have retained substantial genetic variability. These factors alone make the identification of tilapia strains using molecular markers more complex than for inbred crop lines and clonal horticultural varieties. However, at the core population level, DAPC and our SNP panels were used to assign individuals to populations with a high degree of accuracy, particularly in the case of the full DArTseq panel (Table 2). Furthermore, SNP genetic affinities among core breeding populations (Figures 3–5) broadly reflected the documented ancestry of these populations (Figure 1 and Table 1). For example, among Nile tilapia populations, GIFT/GIFT-derived and non-GIFT populations were readily distinguished, with the notable exception of Chitralada. The close SNP genetic affinity of Chitralada with GIFT and GIFT-derived strains—BEST, ExCEL, GIFT-WF, and GIFTFF—seems incongruous, given their putative ancestry (Figure 1), but has been observed in other studies involving similar populations (Moses et al., 2020).

For the majority of hatcheries, the hatchery-identified strain accorded with the DAPC-inferred strain, using the reduced subset of informative SNPs. However, for hatcheries where the hatchery-identified and DAPC-assigned strain did not match, it was not possible to independently verify which was correct. The existence of unregulated and uncertified broodstock supply chains or deliberate or inadvertent misrepresentation of broodstock origin could explain misidentification of strains by hatcheries. Furthermore, the genetic management of tilapia stocks held by hatcheries is highly variable—not all maintain records of the origin of their stocks; some maintain multiple strains but may not maintain them separately (i.e., some maintain strain admixtures); not all adopt appropriate practices to limit inbreeding, and not all routinely obtain new genetically superior stocks from core breeding populations. With respect to possible DAPC misassignment, many of the hatchery populations sampled for our study diverged from core populations numerous generations prior to sampling. Accordingly, sampled hatchery populations had undoubtedly genetically diverged, to varying extents, from their core breeding population/s of origin due to selection, genetic drift, and strain mixing, with unpredictable consequences for the accuracy of our assignment method. Indeed, in some hatcheries, there was substantial variation in the DAPC assignments among the approximately 10 individuals sampled from a putatively single strain—indicating that DAPC assignment, for individual animals sampled from hatcheries, using our method, is not sufficiently accurate for most purposes. Nevertheless, in our study, there was strong concordance between hatchery-identified strain and DAPC-inferred strain in hatcheries where 75% or more of DAPC-assigned individuals were in the modal DAPC-assigned strain (Figure 6). This suggests that our method could be used to accurately assign strain to hatchery populations, in Bangladesh and the Philippines, if only DAPC-assignments from hatcheries that met this 75% “confidence threshold” were accepted. Although increasing the accuracy of assignment, adopting such an approach inevitably results in a substantial proportion of hatcheries being categorized as “unassigned” and does not totally exclude the possibly of false assignments.

In Bangladesh, it was evident that a disproportionate number of hatcheries with an unknown hatchery-identified strain had a DAPC-inferred strain of Chitralada (Table 3). It is conceivable that hatcheries with stock of unknown origin are more likely to hold local strains descended from early introductions of Thai origin (Figure 1) (Hussain et al., 2014). Alternatively, hatcheries may have been unwilling to identify their strain as Chitralada if their broodstock were sourced through informal channels. However, it is also possible that DAPC incorrectly inferred that fish were from the Chitralada strain, given the close SNP genetic affinities between GIFT/GIFT-derived strains and Chitralada (Figures 3, 4).

In our study, DAPC assignment to groups (i.e., GIFT, GIFT-derived, and non-GIFT), rather than individual strains, only marginally improved the concordance between hatchery-identified and DAPC-inferred results (Figure 6). However, single-nucleotide polymorphisms included in our reduced subset of informative SNPs were selected to maximize the ability to distinguish between sampled core populations of key tilapia strains, not groups. This approach likely resulted in ascertainment bias toward SNP affected by selection, or genetic drift, subsequent to the divergence of core populations (e.g., GIFT-WF and GIFTFF, Figure 1). Accordingly—if the only objective of the study had been to distinguish between GIFT, GIFT-derived, and non-GIFT groups, ignoring individual strain—an alternative approach to SNP selection should have been adopted to obtain an optimal subset of informative SNPs for this purpose.

Despite their respective limitations, the hatchery surveys and strain assignment using DAPC confirmed the ongoing importance of GIFT and GIFT-derived strains to tilapia aquaculture in Bangladesh and the Philippines (Gupta and Acosta, 2004; ADB, 2005; Ponzoni et al., 2010). In Bangladesh, the dominant hatchery-identified and DAPC-assigned strains were GIFT-WF or GIFTFF, and in the Philippines, GET-ExCEL—a composite strain partially derived from GIFT (Figure 1)—was the most prevalent. Our study also highlighted the prevalence of locally developed strains in the Philippines and absence of such strains in Bangladesh. The Philippines has a long history of tilapia genetic improvement, beginning with the development of GIFT in the 1980s (Figure 1) and, accordingly, has mature, structured, and systematic genetic improvement, dissemination, and extension programs in place. In Bangladesh, the tilapia sector has expanded rapidly in recent decades, and genetic improvement and associated systems are currently less sophisticated. This distinction was possibly reflected in the higher proportion of hatcheries with an unknown strain in Bangladesh (Tables 3B, 4B), with recent and informal introductions likely to be a factor in the inability of hatchery owners to identify the origins of their stock.

In conclusion, this study (i) successfully identified and characterized single-nucleotide polymorphisms (SNPs) for Nile tilapia; (ii) SNP genetic affinities among core breeding populations were shown to broadly reflect the documented ancestry of these populations, with the notable exception of Chitralada; (iii) identified a subset of SNPs and developed a tool to assign individuals to strains using DArTcap genotyping and DAPC methods; and (iv) found that, in the majority of 205 sampled hatcheries in Bangladesh and the Philippines, the hatchery-identified strain accorded with the DAPC-inferred strain (or group). Furthermore, the study verified the importance of GIFT and GIFT-derived strains to tilapia aquaculture in these countries. However, for hatcheries where the hatchery-identified and DAPC-assigned strain (or group) did not match, it was not possible to independently verify, which was correct, and the possibility of false DAPC assignment could not be excluded. Accordingly, our SNP panel and assignment method must be implemented in a manner that recognizes its inherent limitations—such as excluding hatchery-level DAPC assignments that do not meet a predefined “confidence threshold” —to avoid spurious conclusions.

## Data Availability Statement

The datasets presented in this study can be found in online repositories: https://doi.org/10.7910/DVN/PPOSWW (Hamilton et al., 2020).

## Ethics Statement

Ethical review and approval was not required for the animal study because as stated in the Materials and Methods, fish sampled for this study were handled and biopsied using standard practices routinely employed in commercial tilapia operations. Fish were fin clipped using non-lethal, humane methods in accordance with the Guiding Principles of the Animal Care, Welfare and Ethics Policy of the WorldFish Center (Worldfish, 2004). Written informed consent for participation was not obtained from the owners because fish sampled for this study were sampled in 2015 with the full knowledge and consent of the owners.

## Author Contributions

CL and JB oversaw the project, identified the subset of informative SNPs, and undertook initial analyses and reporting. BB, RV, and MD contributed to the hatchery sampling design and coordination of hatchery samples. MH undertook the final analyses and produced the first draft of the manuscript. All authors reviewed and contributed to the final version of the manuscript.

## Conflict of Interest

The authors declare that the research was conducted in the absence of any commercial or financial relationships that could be construed as a potential conflict of interest.
