# Supplementary material for: Distinguishing Between Nile Tilapia Strains Using a Low-Density Single-Nucleotide Polymorphism Panel
Source: Front Genet. 2020 Dec 1;11:594722. doi: 10.3389/fgene.2020.594722 (PMC7736061; doi:10.3389/fgene.2020.594722)
Supplement: Supplementary file 1 [file Table_1.DOCX]

**Supplementary Materials 1.** Summary statistics for Single Nucleotide Polymorphisms prior to quality control

|  | DArTseq | DArTcap |
| --- | --- | --- |
| Number of markers | 21195 | 1334 |
| Fragments sequenced containing one marker | 15297 | 709 |
| Fragments sequenced with multiple markers | 2702 | 284 |
| Unique fragments | 17999 | 993 |
| Average fragment length (base pairs) | 60.1 (0.10) | 64.9 (0.25) |
| Fragment length minimum (base pairs) | 20 | 28 |
| Fragment length maximum (base pairs) | 69 | 69 |
| Polymorphic information content | 0.20 (0.001) | 0.16 (0.003) |
| Call rate | 0.82 (0.001) | 0.97 (0.001) |
| Reference read depth | 23.3 (0.19) | 152.2 (3.49) |
| SNP read depth | 17.1 (0.15) | 94.2 (2.36) |
| Reproducibility* | 0.992 (0.0001) | 0.989 (0.0004) |
| Avg. missing data per individual (%) | 17.6 (0.22) | 3.2 (0.17) |

* Reproducibility is the proportion of technical replicate assay pairs for which the marker score was consistent.
