# Supplementary material for: Distinguishing Between Nile Tilapia Strains Using a Low-Density Single-Nucleotide Polymorphism Panel
Source: Front Genet. 2020 Dec 1;11:594722. doi: 10.3389/fgene.2020.594722 (PMC7736061; doi:10.3389/fgene.2020.594722)
Supplement: Supplementary file 2 [file Table_2.DOCX]

**Supplementary Material 2: Identification of the ‘full list of informative SNPs’**

When screening for the ‘most informative’ markers across multiple populations, the marker with the greatest value (e.g. $\boldsymbol{F}_{\boldsymbol{ST}}$) overall can potentially be heavily biased by a single, highly differentiated population. In this case, the markers selected can perform well at distinguishing between the highly differentiated population and the others, but can perform poorly in distinguishing the more closely related groups.

In order to overcome this bias, comparisons across all possible pairwise combinations were set up. This dataset comprised of 10 different populations, which equates to 46 unique pairwise combinations.

Two statistics were calculated for individuals from each population pairwise combination:

$${\boldsymbol{F}_{\boldsymbol{ST}}\boldsymbol{=}\boldsymbol{(H}}_{\boldsymbol{T}}\boldsymbol{-}\boldsymbol{H}_{\boldsymbol{S}}\boldsymbol{)/}\boldsymbol{H}_{\boldsymbol{T}}$$

where $\boldsymbol{H}_{\boldsymbol{T}}$ is the expected heterozygosity across the total population and $\boldsymbol{H}_{\boldsymbol{S}}$ is the expected heterozygosity of the of the individual core breeding populations

$$\boldsymbol{\delta}\boldsymbol{=}\left| \boldsymbol{p}_{\boldsymbol{Ai}}\boldsymbol{-}\boldsymbol{p}_{\boldsymbol{Aj}} \right|$$

where $\boldsymbol{p}_{\boldsymbol{Ai}}$ and $\boldsymbol{p}_{\boldsymbol{Aj}}$ are the frequencies of allele A in the *i*^th^ and *j*^th^ core breeding populations, respectively.

The top ranked SNP markers for each pairwise combination were selected based on both $\boldsymbol{\delta}$ and $\boldsymbol{F}_{\boldsymbol{ST}}$.(Table S2.1)

**Table S2.1.** A summary of the number of unique SNP for each metric and cutoff criteria (top ranked SNPs).

| **Metric** | **Top 2** | **Top 5** | **Top 10** | **Top 25** | **Top 50** | **Top 60** | **Top 75** | **Top 100** |
| --- | --- | --- | --- | --- | --- | --- | --- | --- |
| $\boldsymbol{F}_{\boldsymbol{ST}}$ | 45 | 122 | 230 | 506 | 881 | 1057 | 1297 | 1639 |
| $\boldsymbol{\delta}$ | 46 | 120 | 227 | 481 | 842 | 1005 | 1214 | 1538 |
| Combined* | 50 | 133 | 254 | 545 | 962 | 1140 | 1387 | 1742 |

*Pairwise $\boldsymbol{F}_{\boldsymbol{ST}}$ and pairwise $\boldsymbol{\delta}$ combined (i.e. all unique SNP resulting from rows 1 and 2)
