# Supplementary material for: Distinguishing Between Nile Tilapia Strains Using a Low-Density Single-Nucleotide Polymorphism Panel
Source: Front Genet. 2020 Dec 1;11:594722. doi: 10.3389/fgene.2020.594722 (PMC7736061; doi:10.3389/fgene.2020.594722)
Supplement: Supplementary file 3 [file Table_3.DOCX]

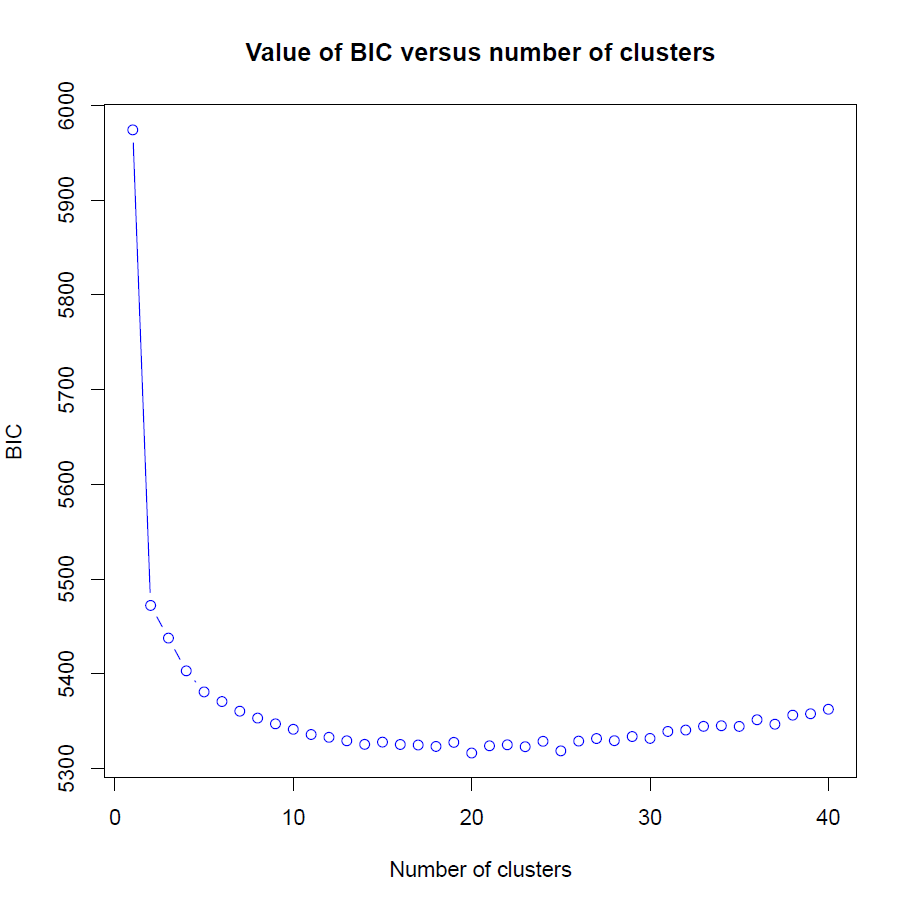

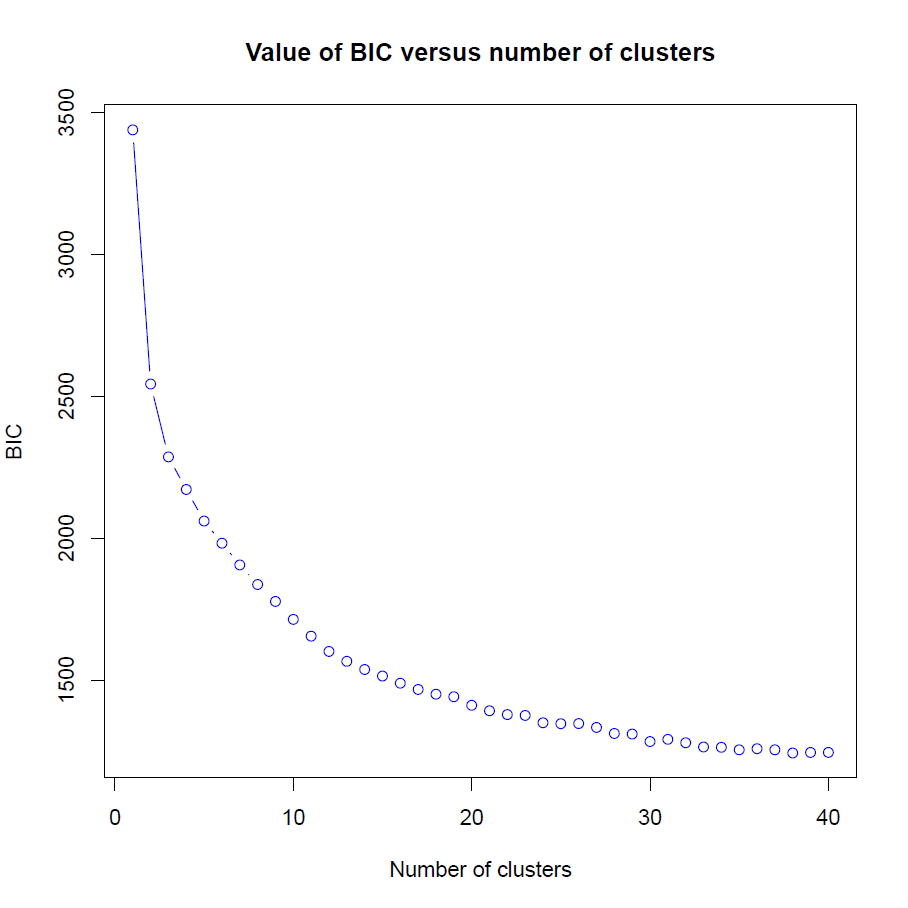


1. **B)**

**Supplementary Materials 3.** Bayesian Information Criterion (BIC) against the number of clusters (k) from unsupervised k-means clustering for (A) the full DArTseq panel and (B) the reduced subset of informative SNPs.
